# Association of Neuroticism Personality According to the Degree of Social Contact with Incident Dementia

**DOI:** 10.1002/alz.086107

**Published:** 2025-01-09

**Authors:** Yufei Liu, Yi Tang, Jie Chang

**Affiliations:** ^1^ Xuanwu Hospital, Capital Medical University, National Center for Neurological Disorders, Beijing, China, Beijing, Beijing China; ^2^ Department of Neurology & Innovation Center for Neurological Disorders, Xuanwu Hospital, Capital Medical University, National Center for Neurological Disorders, Beijing, Beijing China; ^3^ Xuanwu Hospital, Capital Medical University, Beijing, Beijing China

## Abstract

**Background:**

Personality traits, especially neuroticism, can influence susceptibility to dementia. Although social contact can mitigate stress and risk of dementia, the extent to which social contact can mitigate excess risk associated with neuroticism remains unclear. The objective of study was to investigate changes in neuroticism‐associated excess risk of dementia arising from different levels of social contact.

**Method:**

This prospective cohort study examined 393939 UK Biobank participants assessed from 2006‐2010 and followed up until December 2022. Participants without dementia at baseline or the first 2‐year follow‐up, and with complete neuroticism and social contact data were included. Neuroticism was measured using the Revised Eysenck Personality Questionnaire, categorized into low and high neuroticism. Low, intermediate, or high social contact levels were assessed based on household size, contact with family or friends, and group participation. Dementia incidence (all‐cause dementia, Alzheimer’s disease [AD], and vascular dementia [VaD]) were determined using linked electronic health records. Cox proportional hazards regression models estimated hazard ratios (HRs) and 95% confidence intervals (CIs) for dementia risk, adjusted for multiple covariates.

**Result:**

Among 393939 participants (mean [SD] age: 56.4 [8.1] years; 53.7% female), 6588 cases of all‐cause dementia were recorded over 13.7 years. High neuroticism was associated with increased all‐cause dementia risk (HR = 1.16, 95% CI = 1.10‐1.22) and cause‐specific dementia subtypes (AD: HR = 1.10, 95% CI = 1.02‐1.19; VaD: HR = 1.16, 95% CI = 1.05‐1.29). Among high neuroticism participants, excess risk of all‐cause dementia showed a stepwise decrease with increasing social contact (low contact: HR = 1.27, 95% CI = 1.15‐1.40; intermediate contact: HR = 1.20, 95% CI = 1.12‐1.28; high contact: HR = 1.07, 95% CI = 1.00‐1.15). High social contact similarly decreased excess risk of AD (HR = 1.06, 95% CI = 0.95‐1.17) and VaD (HR = 0.96, 95% CI = 0.82‐1.12), comparable to those with low neuroticism.

**Conclusion:**

Enhanced social contact is associated with a stepwise reduction in dementia risk and may mitigate or potentially eliminate excess risk of dementia in individuals with high neuroticism, supporting social contact as a preventive strategy to reduce excess risks of dementia from neuroticism personality trait.

